# Li–Fraumeni Syndrome: Mutation of *TP53* Is a Biomarker of Hereditary Predisposition to Tumor: New Insights and Advances in the Treatment

**DOI:** 10.3390/cancers14153664

**Published:** 2022-07-27

**Authors:** Valentina Rocca, Giovanni Blandino, Lucia D’Antona, Rodolfo Iuliano, Silvia Di Agostino

**Affiliations:** 1 Department of Clinical and Experimental Medicine, Magna Græcia University of Catanzaro, 88100 Catanzaro, Italy; valentina.rocca@unicz.it; 2 Translational Oncology Research Unit, IRCCS Regina Elena National Cancer Institute, Via Elio Chianesi, 53, 00144 Rome, Italy; giovanni.blandino@ifo.it; 3 Medical Genetics Unit, Mater Domini University Hospital, 88100 Catanzaro, Italy; dantona@unicz.it (L.D.); iuliano@unicz.it (R.I.); 4 Department of Health Sciences, Magna Græcia University of Catanzaro, 88100 Catanzaro, Italy

**Keywords:** Li–Fraumeni syndrome (LFS), *TP53*, biomarker, cancer predisposition, germline mutation

## Abstract

**Simple Summary:**

Li–Fraumeni Syndrome (LFS) is a rare tumor predisposition syndrome in which the tumor suppressor *TP53* gene is mutated in the germ cell population. LFS patients develop a broad spectrum of cancers in their lifetime. The risk to develop these tumors is not decreased by any type of treatment and if the analysis of the *TP53* mutational status in the family members was not possible, tumors are often diagnosed in already advanced stages. This review aims to report the evidence for novel mechanisms of tumor onset related to germline *TP53* mutations and possible treatments.

**Abstract:**

Li–Fraumeni syndrome (LFS) is a rare familial tumor predisposition syndrome with autosomal dominant inheritance, involving germline mutations of the *TP53* tumor suppressor gene. The most frequent tumors that arise in patients under the age of 45 are osteosarcomas, soft-tissue sarcomas, breast tumors in young women, leukemias/lymphomas, brain tumors, and tumors of the adrenal cortex. To date, no other gene mutations have been associated with LFS. The diagnosis is usually confirmed by genetic testing for the identification of *TP53* mutations; therefore, these mutations are considered the biomarkers associated with the tumor spectrum of LFS. Here, we aim to review novel molecular mechanisms involved in the oncogenic functions of mutant p53 in LFS and to discuss recent new diagnostic and therapeutic approaches exploiting *TP53* mutations as biomarkers and druggable targets.

## 1. Introduction

Li–Fraumeni syndrome (LFS) was first described in 1969 by Frederick Pei Li and Joseph Fraumeni Jr., who observed in some families a high frequency, even at a young age, of some types of cancer [1]. To date, it is estimated that around one thousand families from 172 different countries are affected worldwide [2]. LFS is a rare autosomal dominant familial cancer syndrome characterized by the early onset of multiple tumors, particularly soft-tissue sarcomas, osteosarcomas, breast tumors, brain tumors, adrenal cortical carcinomas, and leukemia [2,3]. In 1990, David Malkin identified a *TP53* germline mutation closely associated with LFS [4]. Subsequently, the germline mutation of the *TP53* gene was identified in about 70% of LFS families [5,6]. Along with numerous studies linking some *TP53* missense mutations as carriers of gain-of-function oncogenic activities (GOFs) in sporadic (unfamiliar) kinds of cancer, genetic studies in LFS have suggested that inherited *TP53* mutations may be responsible for the increased susceptibility to cancer [7,8,9]. Interestingly, recent genome-wide studies have shown that *TP53* germline mutation carriers have 80% penetrance by age 70, showing the onset of distinct groups of cancers across age groups. For example, in the childhood phase (0–15 years, 22% of all cancers) the most characteristic tumors are adrenal cortical carcinoma, rhabdomyosarcoma, and medulloblastoma; the phase of early adulthood (16–50 years, 51%) includes breast cancer, osteosarcoma, soft-tissue sarcomas, leukemia, astrocytoma, glioblastoma, colorectal, and lung cancer; in the stage of late adulthood (51–80 years, 27%) pancreatic and prostate cancers are the most prevalent [10,11].

Before the diffusion of next-generation sequencing (NGS) into clinical diagnostics, the detection of germline *TP53* mutation has been limited to patients who met clinical criteria for LFS. Multigene panel tests (MGPT) can simultaneously evaluate multiple hereditary cancer genes for cancer predisposition [12,13,14]. The increasing use of multiple genetic panel-based analyses allowed considering mutp53 genetic testing in patients that do not meet the LFS single genetic test criteria (SGT), leading to the detection of *TP53* variants associated with a less penetrating phenotype. This interesting point opened a debate regarding the relationship between the *TP53* genotype and the LFS phenotype. To date, the mechanisms that allow differentiating the phenotypic expression of *TP53* mutations identified by using MGPT from those identified by SGT triggered by classical phenotypic presentations are not known [14,15].

In the past 30 years, there was a lack of a generally accepted definition that reflects the evolving phenotypic spectrum of LFS and, increasingly, individuals with a pathogenic/probable pathogenic variant of *TP53* are found who do not meet the criteria of the LFS single genetic test. In 2001, the Chompret criteria first defined the *TP53* test for childhood cancer cases and patients with early onset breast cancer [16,17]. These criteria are constantly evolving, especially with the recent adoption of MGPT [15,18,19].

The major aim is to find the basis of associations between *TP53* genotype and tumor phenotype and to stratify clinical management depending on *TP53* status. Furthermore, there is no type of cancer exclusively associated with specific mutation classes or peculiar variants of *TP53*. For example, the p53-R337H protein mutation was believed to predispose to adrenocortical carcinoma. However, it has recently been revealed that this mutation is also related to the onset of choroid plexus carcinoma and breast cancer [20,21].

Certainly, in the future NGS approaches in the analysis of tumors from individuals with LFS will make it possible to identify numerous factors such as copy number variations, non-coding differentially expressed RNAs, or others.

## 2. The *TP53* Gene and Role of Mutant p53 Proteins in Cancer

**Figure 1:**
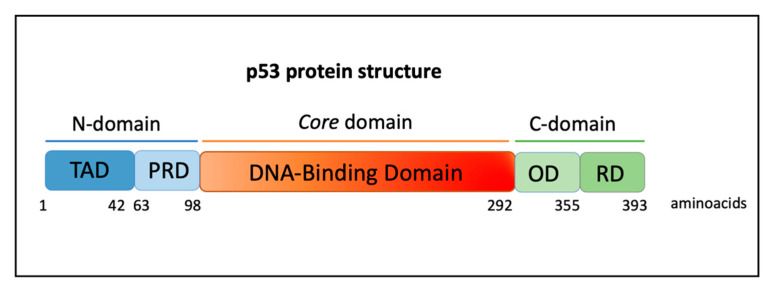
Protein domains of p53. Human p53 protein has 393 aminoacidic residues and is composed of a transactivation domain (TAD), proline-rich domain (PRD), DNA-binding domain (DBD), tetramerization or oligomerization domain (OD), and a C-terminal regulatory domain (RD).

The nuclear phosphoprotein p53 has a long and fascinating history. It was first discovered in 1979 as a transcriptional factor associated with the large T antigen SV40 in virally transformed cancer cells, considered a proto-oncogene able to increase cell growth [22,23,24,25]. The p53 protein works as a tetramer, each monomer consists of an N-terminal transactivation domain (TAD), a proline-rich domain (PRD), a core DNA-binding domain (DBD), a tetramerization domain (OD), and a C-terminal regulatory domain (RD) (Figure 1) [26].

**Figure 2:**
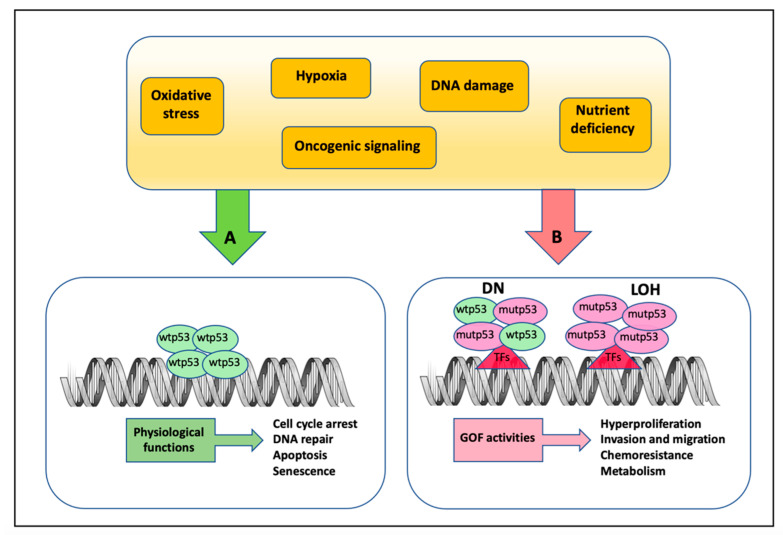
Mechanisms of action of wild-type and mutant p53 protein in response to different stresses. (**A**) In response to diverse types of cellular stress signals, the wild-type p53 protein is induced to activate different pathways by its recruitment onto specific DNA consensus sequences of target gene promoters. (**B**) On the other side, mutant p53 forms oncogenic complexes with other transcription factors, usually not bound by wild-type p53, resulting in the aberrant activation of genes that promote GOF activities. Notably, when *TP53* mutations occur in one of the alleles, mutant p53 co-exists with wtp53 acting as a dominant negative (DN) factor in the heterodimer complexes, until the loss of the wild-type allele by loss-of-heterozygozity (LOH).

After a few years of study, it became clear that p53 acts as a tumor suppressor and it is the most important decision maker of cell fate that earned it the appellation of “the guardian of the genome”. Different sources of stress such as DNA damage, oncogene activation, nutrient deprivation, oxidative stress, and others induce post-translational changes in p53 that lead to its activation, stabilization, and accumulation in the cells (Figure 2) [25,27]. The tumor suppressor activity of p53 is attributed to its transcriptional regulation of genes involved in numerous cellular processes, such as cell cycle arrest, apoptosis, senescence, DNA repair, and cell differentiation (Figure 2) [27].

The *TP53* gene appears to be mutated in about 50% of all human cancers. About 80% of these mutations are missense and produce a full-length, very stable protein that tends to accumulate at high levels in cancer cells [7,9]. Some of these mutations act as a dominant negative (DN) of wild-type p53 (wtp53), affecting tumor-suppressive pathways. Often, loss-of-heterozygozity events (LOH) of wild-type alleles occur [25,27]. The spectrum of *TP53* mutations in human tumors is intriguing for its variegation and tissue specificity. *TP53* is mutated in more than 90% of ovarian cancers while less than 15% in acute myeloid leukemias, suggesting that there may be some tissue-specific requirements [28].

Most of the *TP53* mutations are recapitulated in six “hotspots” in the DBD (R175, G245, R248, R249, R273, and R282) and abrogate the conventional transcriptional activity of the wtp53 protein [29]. Usually, mutations of p53 are divided into two groups: DNA contact mutations, such as p53R248Q and p53R273H, which affect the domains of the protein that are directly involved in DNA binding, and the conformational mutations, such as p53R175H and p53H179R, which cause a distortion of the correct DBD folding of the p53 protein [9,30].

A growing number of studies support the idea that wtp53 activities must be lost to promote tumorigenesis and metastasis [31]. Furthermore, missense mutations determine the acquisition of mup53 GOFs that are exercised in various oncogenic activities such as increased proliferation and invasion, dysregulation of metabolic activities to support tumor progression, drug resistance, induction of genomic instability, and many others, all participating in the patient’s poor prognosis and survival (Figure 2) [9,32,33,34,35,36]. The molecular mechanisms through which mutp53 exerts its novel oncogenic transcriptional deregulation have been the subject of extensive studies and new ones are still discovered today [37,38,39,40,41,42]. Interestingly, different mutp53s appear to have different GOFs by acting with distinct mechanisms depending on the tumor tissue context, suggesting that genetic background and the influence tumor microenvironment could be responsible for some of the inconsistencies in the diverse mechanisms [9,43,44,45,46].

Studies on murine in vivo models provided valuable information on the understanding of mutp53’s mechanisms of action, protein stabilization, and the related cellular pathways that are affected during tumorigenesis and metastasis [47,48,49]. The development of further in vivo models provided insight into the role of the tumor stroma in the events leading to the GOF activities of mutp53 [48,50]. In vivo and mutp53 PDX models could be employed to study the effects of new drugs in addition to the therapies used in clinical trials that are being developed based on the reactivation of mutp53 protein towards wild-type activities [48,51,52,53,54,55].

Currently, at diagnosis, patients are stratified into wild-type and mutant p53; however, we could imagine a functional classification based on the activities of the mutp53 allele that has been characterized in basic research. This could influence the therapeutic choice based on the functional activities of the mutp53 rather than on genetic modification. This approach in the study of sporadic tumors with *TP53* mutations may be translated to the study of the LFS phenotypes.

## 3. Mutational Landscape of *TP53* in LFS

The interpretation of germinal variants associated with genetic diseases was standardized by the guidelines provided by the American College of Medical Genetics (ACMG), based on various criteria that take into consideration a series of features characterizing genetic variants [56]. Recently, these criteria have been refined, tailoring them for classifying *TP53* germline variants [57].

**Figure 3:**
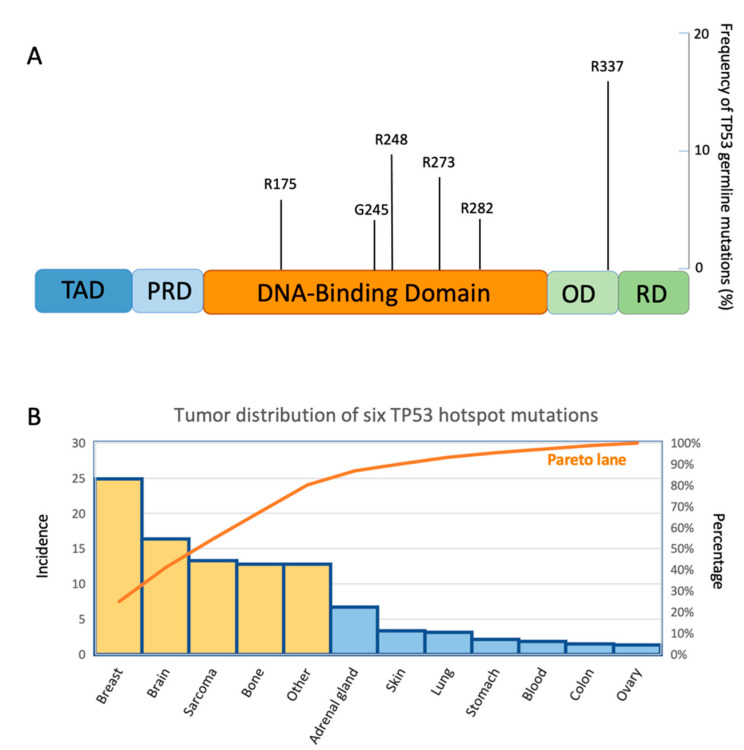
(**A**) A lolliplot showing six mutant p53 germline variants in Li–Fraumeni syndrome with their frequency of mutation. (**B**) Pareto diagram representing the frequency of occurrence of six mutant p53 hotspots in the LFS cancers. The orange bars represent the tumors with a higher percentage of *TP53* mutations according to the Pareto analysis.

In LFS, the distribution of germline *TP53* mutations is similar to that identified in sporadic tumors, with some exceptions in some populations around the world which we will describe later. Many of these mutations are in the DBD and six are hotspots common to those of sporadic tumors: p.Arg175His, p.Gly245Asp, p.Gly245Ser, p.Arg248Gln, p.Arg248Trp, p.Arg273Cys, p.Arg273His, and p.Arg282Trp (Figure 3A) [58,59]. Both in LFS and in sporadic tumors, these hotspots are associated with a poorer prognosis for the patients. However, their interpretation remains challenging. *TP53* variants can act either with a loss of function (LOF) or a DN effect. Nonsense and frameshift variants are easier to interpret because they impair the protein function in an obvious manner, generating a premature stop codon. Although rarer than missense, these variants were found in a significant proportion of patients with LFS [60,61,62]. However, the type and location of the *TP53* variants in LFS, even if they are the same as in sporadic tumors, influence the biological activity of the mutp53 protein and the development of different types of tumors, with differences determined by the family history (Figure 3B) [60]. On the other hand, the penetrance of the disease and the risk of developing secondary tumors are influenced by the type of *TP53* germline mutation. A detailed analysis of 214 LFS families reported that 96% presented point mutations, 67% harbored missense mutations, and 35% dominant-negative mutations, distributed along DBD [60].

The penetrance of truncating variants (frameshift and nonsense) in comparison with missense variants remains debated and still unclear [60,63,64]. Two studies with an extensive genotype–phenotype correlation analysis have been recently published [63,64]. The first one [63] classified *TP53* variants in truncating (with premature stop codon), missense with or without DN, as also previously reported by Giacomelli and colleagues [65], and missense in relevant *TP53* hotspots: p.Arg175His, p.Gly245Asp, p.Gly245Ser, p.Arg248Gln, p.Arg248Trp, p.Arg249Ser, p.Arg273Cys, p.Arg273His, and p.Arg282Trp [58,59]. The results of this study showed that truncating and hotspot variants have higher effects on cancer severity. The second study described *TP53* variants also following the criteria of Giacomelli et al., but considering either possible DN or LOF mechanisms. In both studies, there was a subclass of missense variants that have a small but significant lowering effect on cancer than other variants [63,64], and the presence of a mutp53 LOF could be relevant to increasing the severity of the disease.

The concept that different *TP53* germline alterations confer a preferential association with a tumor phenotype is supported by the evidence that carriers of the founder mutation *TP53* p.R337H in southern Brazil exhibit mainly adrenocortical tumors (ACT) in children, although ACTs are rare cancers [66]. The 377 aminoacidic residues of the p53 protein are located inside the tetramerization domain and it is interesting to point out that this is the only type of mutation so far significantly associated with LFS [60].

However, it has recently been documented that mutp53R337H has a low penetrance, but the aggressiveness of ACT differs depending on the age of the groups in which the tumor has been diagnosed [20]. The predominance of maternal mutated allele inheritance was demonstrated in the LFS Brazilian families. Furthermore, it is very important that in some families, nine malignant neoplasms are diagnosed in asymptomatic carriers by applying the Toronto screening protocol that we will describe in the following chapters [20]. Importantly, additional genetic alteration in ACT includes somatic mutations in ATRX that encodes a helicase that is involved in remodeling and telomere structure maintenance. In Pinto et al.’s study, 37 ACTs were analyzed by WGS and WES, germline *TP53* mutations were present in 25 of the 37 patients (68%). *ATRX* somatic nonsense mutations and SVs deleting multiple exons were identified in 6 of the 19 ACTs (32%), all of which were associated with germline *TP53* mutations. The *ATRX* R2164S somatic missense mutation was also identified by WES in the case with somatic homozygous deletion of *TP53*, both germline *TP53* and somatic ATRX mutations were associated with genomic abnormalities [67].

An interesting study related TP53-R337H with the XAF1 factor. DNA from 203 patients with cancer and 582 relatives were analyzed with NGS and WES. The authors reported that wild-type XAF1 enhances transactivation of wtp53, whereas XAF1 E134* attenuates regulatory activity. In conclusion, haplotype R337H and E134* could modulate cancer phenotype [68].

In addition to the Brazilian-specific mutation, a rare p53 tetramerization domain missense mutation (c.1000G>C; p.G334R) has been identified in nine individuals from four independent family descendants from Ashkenazi Jewish ancestors, suggesting that this is a founder mutation [69]. These individuals were characterized by multiple late-onset LFS-spectrum cancers and available tumors showed biallelic somatic inactivation of *TP53*. The in vitro experiments showed that mutp53G334R fails to transactivate a part of the target genes of wtp53, conferring increased cell proliferation activity and ability to form colonies [69]. These data suggest that descendants of this ethnicity and c.1000G>C carriers should undergo screening and preventive measures to reduce cancer risk.

There are some important gene alterations that influence the LFS phenotype, such as the single-nucleotide polymorphism (SNP) within the first intron of MDM2 (NM_002392.3:c.14+309T>G; SNP309; rs2279744); *TP53* polymorphisms, such as a duplication within intron 3 (PIN3), Pro72Arg; telomere length; differential methylation or variant alleles in miRNAs that modify p53-mediated cell regulation, and the accumulation of copy number variations (CNV) [70]. The impact of the more common Pro72Arg polymorphism remains controversial and MDM SNP309 T>G was shown to be associated with accelerated tumor formation in mutp53 carriers. The results of a French study based on 61 individuals with or without germline mutations in *TP53* from 41 families with LFS confirmed that MDM2 polymorphism had an impact on the age of tumor on set, this effect may be amplified by Pro72Arg polymorphism and the differences between the groups also showed a cumulative effect of these two polymorphisms [71].

Recently, a second MDM2 polymorphism, SNP285G>C (rs117039649), was documented to be in correlation with the SNP309G allele [72,73]. In a large study of 195 LFS patients, this haplotype was shown to influence cancer onset with tumors occurring 5 years earlier [73]. Other genetic factors have been reported as potential modifiers of the LFS phenotype, such as an SNP within the hsa-miR-605 (rs2043556) [74,75].

The extent of the knowledge of how these modifiers consistently influence LFS phenotype remains to be assessed.

## 4. Role of Non-Coding RNAs in LFS: Novel Mechanisms and Hypothesis

To date, MDM2 SNP309 and p53 Arg72Pro polymorphism as well as DNA copy number variations (CNVs) have been associated with cancer risk in LFS further serving as a marker for clinical monitoring of these patients [71,76,77]. Recently, it was highlighted that non-coding RNAs could be useful for the same purpose.

Wt and mut p53 expression have been correlated with the expression of microRNAs (miRNAs), long non-coding RNAs (lncRNAs), and circular RNAs (circRNAs) whose main function is to refine the regulation of a plethora of genes correlated with cell proliferation and metabolism [78,79,80,81,82]. From the point of view of LFS, a common single nucleotide polymorphism (SNP) within miR-605 (rs2043556) was associated with gastrointestinal cancer risk and one of its most important reported functions is to target MDM2 mRNA, one of the major regulators of protein stability of both wt and mut p53 [83,84]. E3 ubiquitin-protein ligase MDM2 establishes a negative feedback loop to limit the function of p53, nevertheless, high levels of miR-605 act to inhibit p53/MDM2 interaction leading to p53 accumulation [83]. Consistent with this, it was reported that miR-605 rs2043556 (G-allele variant) was significantly associated with an earlier mean age of LFS tumor onset compared to the common homozygous AA genotype [74].

Exploring an LFS cohort of 238 Brazilian individuals carrying *TP53* p.R337H, miR-605 rs2043556 G allele was present in 57.1% of patients where 10.5% were homozygous [75]. Interestingly, in this cohort of LFS patients, the presence of miR-605 rs2043556 was significantly associated with the onset of multiple primary tumors [75].

These findings, which should be further investigated into different aspects of the LFS phenotype and genotype, strongly suggest that SNP-mediated miR-605 deregulation could affect cancer risk in LFS by inducing some changes in the p53/MDM2 levels. Furthermore, they shed light on a new scenario where non-coding RNA modifiers exist and could act to predispose *TP53* mutation carriers to a spectrum of susceptibilities in LFS.

Recently, it was reported that the mutp53/HIF1α/miR-30d axis induced several alterations of secretory pathway components in sporadic breast cancer, such as the increase of the endoplasmic reticulum membranes, increase in the number of COP-I and COP-II vesicles, stabilization of microtubules, and vesicular-tubulation of the Golgi apparatus [85]. Together, these modifications influenced the secretion of soluble factors and the deposition and remodeling of the extracellular matrix (ECM), affecting the signaling network of the tumor microenvironment [85]. In the same paper, the authors have also shown that in adult primary fibroblasts from two LFS *TP53* p.R248Q patients, mutp53 expression was associated with higher HIF1α and miR-30d levels, and higher protein secretion, compared to fibroblasts from two wtp53 healthy donors [85]. These results sustain the fascinating hypothesis that the mutp53/miR-30d axis leads to increased secretion of ECM remodeling factors playing a role in promoting cancer progression and metastasis via an altered secretome also in LFS.

While in sporadic tumors, the search for predictive biomarkers of the disease or the efficacy of the therapy is constantly being explored in the field of non-coding RNA, not only in tumor tissues and metastases, but also in fluids such as blood, saliva, and urine, after the aforementioned studies, this kind of research never blossomed in the field of LFS. In contrast, in recent years, the number of papers linking mutp53 with non-coding RNA signatures, traced from liquid biopsies of cancer patients and endowed with the predictive power of disease and/or efficacy of therapies is increasing [86,87,88].

Up until today, we have learned that the dysregulation of non-coding RNA interactions within cancer molecular pathways contributes to neoplasia and shows important novel druggable targets. In sporadic cancer, the onset of a gene mutation often does not involve just the mutated gene but can also destroy the non-coding RNA networks that change the balance of the downstream targets of those ncRNAs.

It would therefore be very advantageous to identify any non-coding RNAs that are correlated with Li–Fraumeni tumor genotypes and phenotypes both for basic knowledge and for implementing better therapeutic and active surveillance strategies.

## 5. Tumor Prevention and Treatments

Approximately one thousand multigenerational families worldwide have been estimated to be affected by LFS with no ethnic or geographic disparity in occurrence, although an exceptionally high prevalence of LFS has been documented in Southern and Southeastern Brazil [15,89]. Disease onset is known to commonly occur in children and young adults who develop several multiple cancers, most notably soft-tissue and bone sarcomas [18]. The proportion of individuals with a de novo germline *TP53* pathogenic variant is estimated to be between 7% and 20% [3]. Importantly, not all individuals who have mutp53 will develop cancer, but the risks are significantly higher than in the general population. Once a patient has been diagnosed with LFS, it is important that all family members have genetic counseling and surveillance for early cancer [3,70].

What are the criteria used to decide whether to ascertain that an individual (proband) has LFS? There are diverse published criteria to test individuals for a heterozygous germline pathogenic variant in *TP53*, including the most recently revised Chompret criteria [17,60]. In a proband, the classical LFS diagnosis is established following three fundamental criteria: the diagnosis of sarcoma before the age of 45; a first-degree relative, (a parent, sibling, or child) with any cancer before age 45; a first-degree relative or second-degree relative, meaning a grandparent, aunt/uncle, niece/nephew, or grandchild, with any cancer before age 45, or a sarcoma diagnosed at any age [90]. Recently, the Chompret Criteria have been strongly proposed to identify affected families beyond the classic criteria described above. A diagnosis of LFS and testing *TP53* gene mutation is considered for anyone with a family history that meets one of the following three updated criteria which can be read in a detailed way in Frebourg et al. [90]. Importantly, diagnostic criteria assessed by Chompret increased the sensitivity of *TP53* pathogenic germline variants detection by including patients with core LFS tumors even without a family history. Classic and Chompret criteria together increased the diagnostic sensitivity to 95%, therefore, the National Comprehensive Cancer Network (NCCN) and other guidelines recommend using both the Classic LFS criteria and the revised Chompret criteria to recommend *TP53* germline genetic testing [60,91].

Li–Fraumeni-like syndrome (LFL) is a pathology related to LFS where a set of criteria has been drawn up for affected families who do not meet classic criteria [90]. There are two suggested definitions for LFL called the “Birch” and “Eeles” definitions that introduce the concept of a Li–Fraumeni spectrum that includes the detection of variants with lower penetrance, a broader classification of hereditary cancer-related syndromes *TP53* (hTP53rc), and those who have “phenotypic LFS”, that means patients who show cancer incidence and the family history but without a known genetic driver [16,90,92,93].

Unfortunately, there is currently no specific treatment or standard guidelines for LFS or specific therapy for a variant of the germline *TP53* gene. Therefore, as a rule, the types of tumors are evaluated from time to time and the therapies used for classic cancer patients are adopted. Several studies have shown that active surveillance of LFS patients is significantly associated with improved survival by tracking presymptomatic malignancies compared to LFS patients who were diagnosed with cancer because the symptoms arose [15,94,95,96,97]. Overall, these studies propose clinical surveillance protocols using physical examination and frequent biochemical and imaging studies for instance consisting of whole-body magnetic resonance imaging (MRI), specific MRI of brain breast, mammography, abdominal and pelvic ultrasound, and colonoscopy. All these very important findings should be material for preparing a common LFS surveillance and treatment registry, from genetic counseling to active surveillance.

Diverse associations of families with LFS have been formed in some countries to inform as much as possible about this rare hereditary disease. We can mention the LFSA (Li–Fraumeni Syndrome Association) in the US, the George Pantziarka TP53 Trust in the UK, or hospitals all over the world that are highly specialized in the detection of germline *TP53* mutations and in the active surveillance of members of the families involved such as the team at the University of Toronto which has developed guidelines, “The Toronto Protocol”, which have proved effective for early detection [94].

Therefore, over the years, scientists have focused their studies mainly on two areas of intervention, namely, on the one hand, the elucidation of the mechanisms underlying the relationship between specific variants of *TP53*, the penetrance and other epidemiological variables, the time of onset and incidence of tumors, and, on the other hand, the experimentation of preventive therapies to avoid the risk of cancer onset. Many of these therapeutic interventions to decrease the incidence of cancer in LFS are based in part on the development of therapies in sporadic cancers expressing high levels of mutp53, such as high-grade ovarian cancer, triple-negative breast cancer, colorectal cancer, or squamous head and neck carcinoma [98,99,100,101]

Some studies reported that the use of radiotherapy is associated with the onset of radiation-induced tumors in LFS patients. Furthermore, many patients are still resistant to radiotherapy, a condition conferred by the gain-of-function activity conferred by some p53 mutations [102,103]. For this reason, when not strictly necessary, computed tomography scans and other diagnostic techniques involving ionizing radiation should be limited.

Interestingly, some clinical trials have been launched in LFS patients, not only with the aim of obtaining results from a therapeutic point of view but also with the aim of exploring drug candidates for cancer prevention in people with LFS (https://clinicaltrials.gov/ct2/home accessed on 15 June 2022).

To date, seventeen studies are reported on ClinicalTrial.gov accessed on 15 June 2022, nine of which are in the recruitment phase, and only one completed with the results available. This latest study reported encouraging data regarding the use of nicotinamide riboside (NR) (vitamin B3) as a dietary supplement in LFS patients (NCT03789175) to improve the mitochondrial function of skeletal musculature and respiratory functions [104].

Among these clinical trials, “A Pilot Study of Metformin in Patients With a Diagnosis of Li-Fraumeni Syndrome” (NCT01981525) supported by the National Cancer Institute (NCI), proposed the repositioning of an existing drug [105]. Metformin (Met) is the most commonly used drug for type 2 diabetes mellitus, it is inexpensive, safe, and efficient in ameliorating hyperglycemia and hyperinsulinemia. Several epidemiological studies have shown that diabetic patients treated with Met have a lower risk of developing cancers [106]. There are therefore many clinical trials that have used Met as an adjuvant in the treatment of many types of cancer [107,108]. From the point of view of the molecular mechanism, Met can affect a large number of molecules and cellular signalings such as influencing gluconeogenesis, metabolic pathways, and mitochondrial cellular respiration [109].

The NCT01981525 trial starts from the experimental data that Met inhibits oxidative phosphorylation, reducing available energy for cancer cell proliferation, to assess the safety and tolerability of Met in nondiabetic LFS patients and to evaluate the modulation of metabolic profiles compared to LFS patients who have not taken the drug [105]. The results of this study reported excellent tolerability of Met and the suppression of hepatic mitochondrial function as expected in these individuals. Updates on the possible onset of tumors in the two groups of patients are desirable in the future. Similar trials in Canada, Germany, and the United Kingdom are planned with the idea of following the groups of patients treated and not treated with Met for 5 years and monitoring the onset of tumors (https://www.oncology.ox.ac.uk/clinical-trials/oncology-clinical-trials-office-octo/prospective-trials/mili accessed on 15 June 2022).

Interestingly, genomic profiling of tumors is becoming a routine practice in translational research and is useful to identify actionable molecular targets for personalized medicine [110,111].

These findings inspired a new approach where the use of a comparative transcriptomic analysis identified a group of genes precisely overexpressed in LFS glioblastoma multiforme (GBM) patients among a cancer compendium of 12.747 tumor RNA sequencing data sets, including 200 GBMs, opening the way for the identification of personalized drug treatments [112]. STAT1 and STAT2 genes are identified to be overexpressed in LFS patients and ruxolitinib was the JNK inhibitor, which could be used as a potential therapy to block the JNK/STAT1,2 pathway [112]. The research of actionable targets by using genomic and transcriptomic approaches and patient-derived organoids could represent the basis of many therapeutic trials in cancer.

## 6. Conclusions

The close connection between LFS and the *TP53* germline mutations has made this hereditary cancer syndrome a unique and useful paradigm for the study of mutated p53 proteins. Furthermore, much has been learned about the molecular mechanisms of action of mutp53 in sporadic tumors. However, it is impossible to predict in carriers of the mutation when the tumors will arise and how to treat these LFS people.

Certainly, collaborative studies between different disciplines that integrate clinical practice, and the results of genomic, metabolomic, and biological analyses will be advantageous in developing the understanding of this cancer predisposition syndrome to find coding pathways.

This information may be an important source for explaining the different manifestations of LFS, such as differences in disease and penetrance and the heterogeneity of the tumors that arise. The dream would be to identify the driving factors of the phenotype of the disease to fine-tune the development of an individualized monitoring protocol.
